# Hyaluronic Acid and Galacto-Xyloglucan Eyedrop Efficacy in Young-Adult Oral Contraceptive Users of Childbearing Age

**DOI:** 10.3390/jcm11154458

**Published:** 2022-07-30

**Authors:** José-María Sánchez-González, Concepción De-Hita-Cantalejo, María Carmen Sánchez-González

**Affiliations:** Department of Physics of Condensed Matter, Optics Area, Vision Sciences Research Group (CIVIUS), Pharmacy School, University of Seville, 41004 Seville, Spain; mhita@us.es (C.D.-H.-C.); msanchez77@us.es (M.C.S.-G.)

**Keywords:** dry eye disease, hyaluronic acid, galacto-xyloglucan, childbearing age, oral contraceptives, tear film, eyedrops

## Abstract

To assess the efficacy of 0.4% hyaluronic acid and 0.2% galacto-xyloglucan for the subjective symptoms of dry eye disease and tear film invasive and noninvasive signs in 34 young-adult oral contraceptive users of childbearing age, a prospective, longitudinal, single-blind, clinical study was performed in a population of childbearing-age oral-contraceptive consumers. Subjective dry eye disease questionnaires, and invasive and noninvasive tear film assessments were reported before and after six weeks of hyaluronic acid with galacto-xyloglucan (HA-GX) treatment versus hyaluronic acid alone (HA). HA-GX treatment resulted in a greater decrease in the ocular surface disease index (17.01 ± 11.36 score points, *p* < 0.01) than the HA variation (11.61 ± 11.18 score points, *p* < 0.01). The standard patient evaluation of eye dryness also decreased more in the HA-GX group (4.06 ± 5.50 score points, *p* < 0.01) than in the HA alone group (0.70 ± 3.16, *p* = 0.21). Regarding noninvasive break-up time (NIBUT), the HA-GX group’s first NIBUT achieved an increase of 1.75 ± 1.16 s, *p* < 0.01, while the HA-alone group increased by only 0.54 ± 1.01 s, *p* < 0.01. The HA-GX group’s mean NIBUT reported an increase of 3.72 ± 5.69 s, *p* < 0.01; however, the HA-alone group achieved 2.19 ± 5.26 s, *p* = 0.05. Hyaluronic acid in combination with galacto-xyloglucan significantly decreased subjective dry eye disease symptoms and increased first and mean NIBUT compared to hyaluronic acid alone. Galacto-xyloglucan added efficacy in young-adult childbearing-age oral contraceptive users.

## 1. Introduction

Dry eye is a multifactorial disease with various symptoms that include stinging, burning, sensations of dryness and grittiness, and fluctuations in blurred vision and visual acuity [1,2]. The most common cause is hormonal alteration, leading to the poor function of the lacrimal glands and decreased tear secretion [3,4]. Dry eye is more common in women than in men, especially in the menopausal and postmenopausal periods [5]. The tissues that make up the anterior surface of the eye, cornea, conjunctiva, lacrimal gland, and meibomian glands are sensitive to changes in estrogen and progesterone [4,6]. There appear to be relationships between dry eye symptoms and sex steroid hormones, estrogens, androgens and progestins [4].

The meibomian glands have receptors for estrogen and progesterone, and the activation of these receptors modulates secretion [7]. Estrogens and progesterone negatively influence the production of lipids, generating dry eye by evaporation. The influence of progesterone on the meibomian gland is much less than that of estrogen [8,9]. Furthermore, estrogen and progesterone influence the function of the lacrimal gland [10], although authors’ opinions on this are controversial. There have been studies affirming that hormones promote the inflammation of the lacrimal gland [11], while other authors have suggested an anti-inflammatory effect [12,13]. Estrogens also increase the expression of proinflammatory genes in the corneal–conjunctival epithelium [14]. There is evidence of a link between dry eye symptoms and the administration of hormonal contraceptives [15,16], which can be composed of estrogen and progesterone or only progesterone [17]. Their use is widespread among women worldwide in the form of implants, injections, oral administration, skin patches, or intrauterine devices [18].

To alleviate dry eye in women undergoing contraceptive treatment, the use of artificial tears that can be a substitute for the tear film is recommended. All of these substitutes have an aqueous base to which different molecules are added. Hyaluronic acid (HA) is a high-molecular-weight polymer with optimal lubricating properties because of its high capacity to retain water. As a result, it has an effective moisturizing and stabilizing effect on the tear film. In addition, it has an antioxidant, cytoprotective effect and a high regenerative and anti-inflammatory capacity in corneal epithelial cells [19,20]. The concentrations in the marketed formulations vary from 0.03%, 0.1%, 0.15%, and 0.18% to 0.4% [21,22]. However, the effectiveness of hyaluronic acid decreases over time. A cross-linked HA formulation has been suggested [20], as well as combination with other molecules that improve the bioavailability of HA [23].

Galacto-xyloglucan is a glucose polymer obtained from the seeds of *Tamarindus indica* [24]. It has branched side chains as a result of the insertion of a xylose. These xyloses can be linked to a galactose and a fucose. The resulting molecular structure yields the polymer properties that mimic the natural mucosal barrier, protecting the epithelium, and thus limiting the entry of harmful agents [25]. Rolando and Valente [26] showed in their study how dry eye symptoms improved in a group of patients treated with formulations of tamarind seed polysaccharide (TSP) at 0.5% and 1%. The combination of HA and galacto-xyloglucan generates a synergistic action on the anterior surface of the eye that protects it from visual stress as well as environmental and mechanical factors [27]. Uccello-Barretta et al. [23] concluded that a minimum concentration of 1.5 mg/mL of each polymer is required to achieve a stable combination.

The purpose of our research is to assess the efficacy of 0.4% hyaluronic acid and 0.2% galacto-xyloglucan on the subjective symptoms of dry eye disease and tear film invasive and noninvasive signs in young-adult childbearing-age oral contraceptive users.

## 2. Materials and Methods

### 2.1. Design

We conducted this prospective, longitudinal, single-blind, single-center study at the Optics and Optometry cabinets of the Pharmacy School (University of Seville, Seville, Spain). This research was conducted according to the Helsinki Declaration and was approved by the Ethical Committee Board of the University of Seville.

### 2.2. Subjects

All of the included subjects read and signed an informed consent form. An informative sheet was provided to all of the subjects with the detailed study procedure. The inclusion criteria were as follows: (1) childbearing-age oral contraceptive users; (2) age between 18 and 25 years old; (3) ocular surface disease index (OSDI) score greater than 0 points; (4) invasive break-up time (BUT) less than 15 s; (5) completion of all examination procedures; and (6) full comprehension of the sense of this research study and signing of an informed consent form before the measurements. The exclusion criteria were as follows: (1) any previous eye surgery; (2) any systemic diseases; and (3) contact lens wear.

### 2.3. Materials

Noninvasive tear film analysis was performed with the Integrated Clinical Platform (ICP) Ocular Surface Analyzer (OSA) from SBM System^®^ (Orbassano, Turin, Italy). The OSA includes a full assessment of the ocular surface through a combination of dry eye disease diagnostic tests. The instrument is fit in the slit lamp tonometer hall. Among the technical data, the image resolution was 6 megapixels; the acquisition mode was multishot and movie acquisition; the focus could be manual or automatic; Placido disc and NIBUT grids that were available, colored, and sensitive to infrared cameras were accessible; and the light source was infrared LED or blue with LED. Two subjective dry eye disease questionnaires were used: the Ocular Surface Disease Index (OSDI) and the Standard Patient Evaluation of Eye Dryness (SPEED) test.

Regarding the lubricants studied, eyedrop A (hyaluronic acid and galacto-xyloglucan, HA-GX group) was 0.40% hyaluronic acid sodium salt, 0.20% galacto-xyloglucan (extracted from tamarind seed), mannitol, trisodium citrate dihydrate, citric acid monohydrate, and isotonic buffered solution with a sufficient quantity for 100 mL (Aquoral Forte^®^, distributed by ESTEVE Pharmaceuticals^®^, Barcelona, Spain, and manufactured by Omisan Farmaceuti^®^, Guidonia Montecelio, Italy). This eyedrop was packaged in a multidose 10-mL bottle. In the control group, eyedrop B (hyaluronic acid, HA group) was 0.40% hyaluronic acid sodium salt; distilled waters of ginkgo biloba, cranberry, fennel, and spark asiatica; boric acid, sodium tetraborate; and sodium chloride with a sufficient quantity for 100 mL (Eyestil Plus^®^, SIFI, Lombardia, Italy). This eyedrop was packaged in a multidose 10 mL bottle.

### 2.4. Examination Procedure

In the first phase, subjects were included or excluded according to previously defined criteria. Subjects were randomized and divided according to simple and computer-generated random numbers to groups receiving eyedrops A and B. All subjects were instructed to avoid using any lubricants or drops for one week prior to the study. After this, the wash-out period was finished, and subjective questionnaires and a noninvasive examination with OSA were administered, from minor to major tear film fluctuations, in the following order: [1] limbal and bulbar redness classification (LBRC) that detected the blood vessel fluidity of the conjunctiva, evaluating the redness degree with the Efron Scale (0 = normal, 1 = trace, 2 = mild, 3 = moderate and 4 = severe); [2] lipid layer thickness (LLT) evaluation with optic interferometry, evaluating the quantity of lipids layering in 7 different pattern categories (<15 nm—Not present, ~15 nm—Open meshwork, ~30 nm—Close meshwork, ~30/80 nm—Wave, ~80 nm—Amorphous, ~80/120 nm—Color fringes, ~120/160 nm—Abnormal color); [3] tear meniscus height (TMH) measurement evaluating the aqueous layer quantified within a millimeter caliper (≤0.20 mm—abnormal and >0.20 mm—normal); and [4] first and mean noninvasive break-up time (FNIBUT and MNIBUT), evaluated with a special grid cone, which evaluates the quality of the mucin layer in seconds (<10 s—abnormal and >10 s—normal) [28,29].

In a second phase, the subjects were re-evaluated after six weeks with a twelve-hour posology to quantify the ocular surface parameters and subjective questionnaires. Finally, after a rest period of 30 min, an invasive tear film examination was performed with a fluorescein break-up time test. The temperature and humidity of the examination room were stable during all measurements.

### 2.5. Statistical Analysis

Statistical analysis was performed with SPSS statistical software (version 26.0, IBM Corp., Armonk, NY, USA). Descriptive analysis was performed with the mean ± standard deviation (SD) and (range value). The normality distribution of the data was assessed with the Shapiro–Wilk test. Differences in qualitative variables were assessed with the chi-square test. The differences among the first, second and third OSA measurements were performed with Wilcoxon’s test. Differences within both eyedrop groups were analyzed with the U test. The correlation study was evaluated with Spearman’s rho test. For all tests, the level of significance was established at 95% (*p* value < 0.05). The sample size was evaluated with the GRANMO^®^ calculator (Institut Municipal d’Investigació Mèdica, Barcelona, Spain; version 7.12). A two-sided test was used. The risks of alpha and beta were set at 5% and 20%, respectively. The estimated standard deviation (SD) of the differences was set at 7.47 (based on Serrano-Morales et al.’s [22] SD of the main variable), the expected minimum OSDI difference was set at 8 score points, and finally, the loss to follow-up rate was set at 0.10. This procedure achieved a recommended sample size of 32 subjects.

## 3. Results

**Figure 1:**
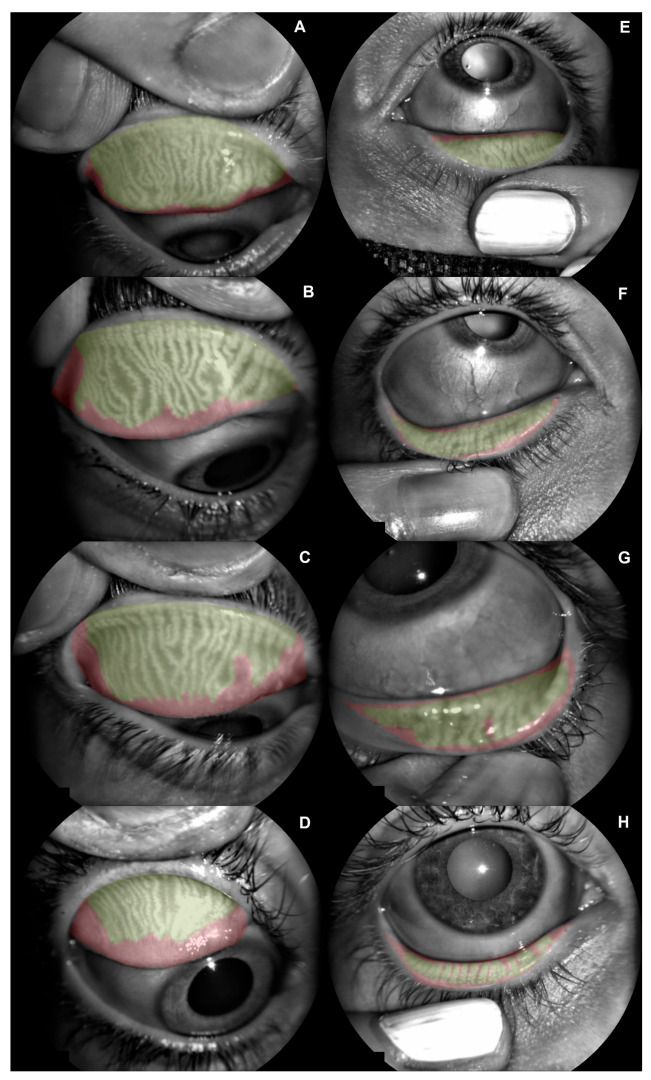
Meibomian gland dysfunction (MGD) distribution among the sample. (**A**) Superior eyelid with mild grade 1 (10.4% of MGD). (**B**) Superior eyelid with severe grade 1 (19.9% of MGD). (**C**) Superior eyelid with mild grade 2 (26.6% of MGD). (**D**) Superior eyelid with severe grade 2 (40.3% of MGD). (**E**) Inferior eyelid with mild grade 1 (11.9% of MGD). (**F**) Inferior eyelid with severe grade 1 (23.7% of MGD). (**G**) Inferior eyelid with mild grade 2 (27.6% of MGD). (**H**) Inferior eyelid with severe grade 2 (45.3% of MGD).

Sixty-eight eyes from thirty-four patients were included in this study. Demographic data about sex distribution, age, sphere refraction, cylinder refraction, axis refraction, CDVA (Decimal Scale), superior and inferior meibomian gland dysfunction percentage in the previous and posterior BUT, OSDI, and SPEED are presented in Table 1. The distribution of superior and inferior eye meibomian gland dysfunction is presented in Figure 1.

### 3.1. Subjective Questionnaires and Break-Up Time

**Figure 2:**
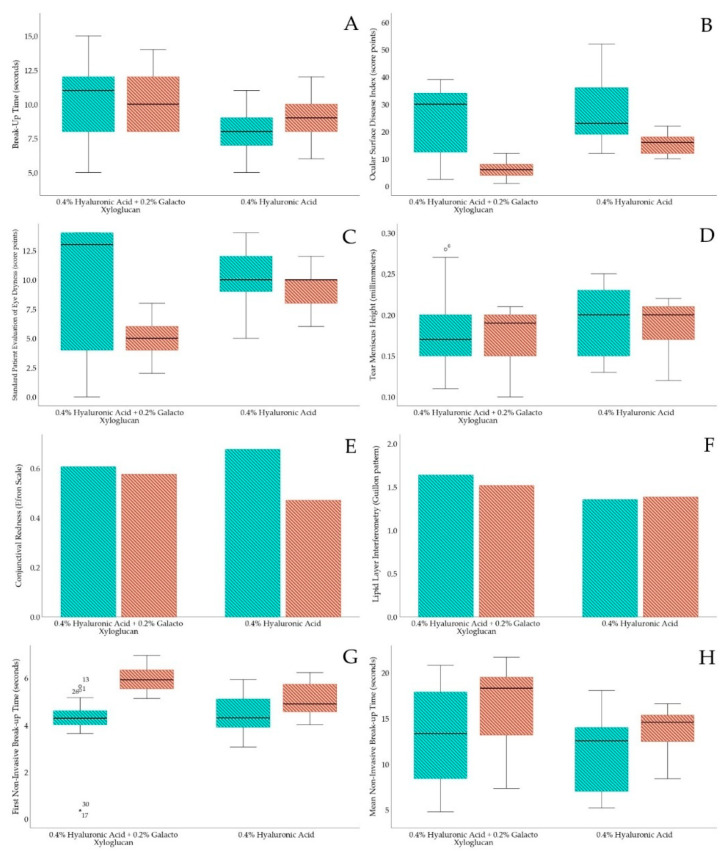
Box and plot longitudinal differences within both eyedrop groups. Green color strip represents previous to treatment and orange strip color represents after treatment. (**A**) BUT test, (**B**) OSDI questionnaire, (**C**) SPEED questionnaire, (**D**) TMH measurement, (**E**) conjunctival redness, (**F**) lipid interferometry, (**G**) first NIBUT and (**H**) mean NIBUT.

The OSDI and SPEED differences between the HA-GX group and HA-alone group within the previous and posterior treatments are presented in Table 1. In a longitudinal approach, for the 0.40% HA 0.20% GX group, the changes between the previous and posterior OSDI questionnaire reported a decrease of 17.01 ± 11.36 score points (*p* < 0.01). Regarding the HA-alone group, the OSDI reported a decrease of 11.61 ± 11.18 score points (*p* < 0.01). According to the SPEED test, the HA-GX group achieved a decrease of 4.06 ± 5.50 score points (*p* < 0.01), and the HA-alone group achieved a decrease of 0.70 ± 3.16 score points (*p* = 0.21). According to the BUT test, the HA-GX group achieved an increase of 0.51 ± 4.43 s (*p* = 0.53), and the HA-alone group also showed an increase of 0.76 ± 2.57 s (*p* = 0.09). Box and plot longitudinal differences between both groups are presented in Figure 2.

### 3.2. Tear Film Noninvasive Tests

Conjunctival redness, lipid layer interferometry, tear meniscus height assessment, and first and mean NIBUT differences between the HA-GX and HA-alone groups are presented in Table 2. With a longitudinal approach, conjunctival redness in the HA-GX group changed only 0.03 ± 0.52 grades on the Efron Scale (*p* = 0.73), similar to that in the HA-alone group, which changed 0.20 ± 0.53 grades (*p* = 0.05). HA-GX group lipid layer interferometry only changed 0.12 ± 1.16 degrees on Guillon Pattern (*p* = 0.55), similar to the HA-alone group, which changed 0.02 ± 1.21 degrees on Guillon Pattern (*p* = 0.81). The TMH of the HA-GX group remained uniform, with a change of 0.01 ± 0.05 mm (*p* = 0.45), similar to that of the HA-alone group, which changed 0.00 ± 0.03 mm (*p* = 0.50). Finally, regarding first and mean NIBUT, the HA-GX group FNIBUT achieved an increase of 1.75 ± 1.16 s (*p* < 0.01), while the HA-alone group increased only 0.54 ± 1.01 s (*p* < 0.01). In addition, the HA-GX group MNIBUT reported an increase of 3.72 ± 5.69 s (*p* < 0.01); however, the HA-alone group achieved 2.19 ± 5.26 s (*p* = 0.05). Box and plot longitudinal differences between both groups are presented in Figure 2.

## 4. Discussion

In our study, the anterior segment of the eye was evaluated with the ocular surface analyzer (OSA) [30,31]. The results show an improvement in subjective symptoms and noninvasive break-up time in a young-adult childbearing population using oral contraceptives after using eyedrops with 0.40% hyaluronic acid and 0.20% galacto-xyloglucan for a period of 6 weeks. Contraceptives consist of a combination of estrogens and progestogens, in most cases synthetic, which inhibit the production of these hormones, inhibiting them via ovulation [32]. There is evidence of the influence of these hormones on the anterior surface of the eye [4,6]. The lipid layer is the superficial and outermost part of the tear film and is responsible for reducing evaporation and keeping the eye moist [33]. Estrogens and progestogens directly affect the functioning of the meibomian glands by altering the production of lipids [8,9], a situation that causes symptoms related to evaporative dry eye, including a sensation of dryness, itching, burning, and limbal and bulbar redness as a consequence of vasodilation of the blood vessels of the conjunctiva and sclera [6].

Our results did not show a decrease in bulbar redness scores in either group, HA-GX or HA. There were also no significant differences in LLT or TMH. The women included in the study were taking oral contraceptives that negatively influence lipid production, generating dry eye by evaporation. Currently, it is very common to include lipids in the composition of artificial tears since their efficacy was shown to improve LLT and reduce tear film evaporation [34,35,36,37]. In our study, no eye drops contained lipids in their composition, and this situation might explain why there were no changes in LLT or an increase in TMH. The innermost mucin layer of the tear film is a thin glycoprotein layer that is highly hydrated and closest to the cornea. The mucin in this layer comes almost entirely from the secretion of goblet cells in the conjunctiva, the function of which is to hydrate and lubricate the anterior surface of the eye [38,39,40]. In the tear evaluation, we included the measurement of two tear breakup times, the first breakup time (FNIBUT) and the mean breakup time (MNIBUT), the latter of which is the average of all tear film breakups that occur throughout the cornea.

**Figure 3:**
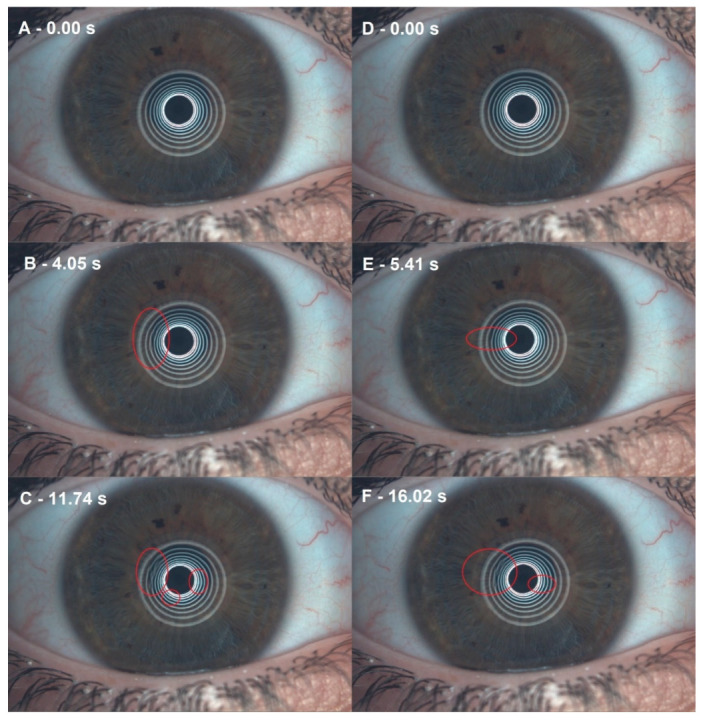
Noninvasive break-up time (NIBUT) differences before and after 0.40% hyaluronic acid with 0.20% galacto-xyloglucan (HA-GX). (**A**) Before HA-GX treatment, the first moment after blinking. (**B**) Before HA-GX treatment, the first NIBUT. (**C**) Before HA-GX treatment, mean NIBUT. (**D**) After HA-GX treatment, first moment after blinking. (**E**) After HA-GX treatment, first NIBUT. (**F**) After HA-GX treatment, mean NIBUT.

In a longitudinal approach, the results show an increase in FNIBUT in both groups, being higher in the HA-GX group (1.75 ± 1.16 s, *p* < 0.01) and lower in the HA group (0.54 ± 1.01 s, *p* < 0.01) but with a statistical significance in both cases. For MNIBUT, the HA-GX group also showed a statistically significant increase (3.72 ± 5.69 s, *p* < 0.01) after six weeks of treatment, while in the HA group, only the mean time increased (2.19 ± 5.26 s, *p* = 0.05) (Figure 3). Furthermore, in our study, we invasively measured BUT with fluorescein, as in the study by Barabino et al. [41], and it was significantly higher in the HA-GX group and lower in the HA group. The reason for these results could be the similarity among the structures of galacto-xyloglucan, the tamarind seed polysaccharide (TSP), and the mucin MUC1 present in the conjunctival epithelium [26,42]. There have been studies showing how the combination of HA-GX is effective in the treatment of dry eye due to its mucoadhesive and mucomimetic properties [24,43,44]. Our results coincide with those of Molina et al. [27].

Furthermore, we used questionnaires to classify the degree of dry eye according to its symptoms, OSDI score, and SPEED score. In a longitudinal approach, our results showed a statistically significant decrease in OSDI scores in the HA-GX group after six weeks of treatment. This trend corresponds to that shown by several studies using the same tear (HA-GX) to assess dry eye symptoms [27,28,43,45]. Other studies have also shown a decrease in the OSDI score when using tears that combine HA with other molecules [43,44,45,46,47,48]. The SPEED score also showed a statistically significant improvement at the end of the study in the HA-GX group. The improvement in symptoms reported by the patients is a consequence of the joint action exerted by HA [19,20] and galacto-xyloglucan [25].

Regarding strengths and limitations, this clinical study involved, to the best of our knowledge, the first evidence of the efficacy of two eyedrops in oral contraceptive users of childbearing age. In addition, noninvasive ocular surface analyzer measurements were used. Regarding limitations, the sample size and follow-up of the research could be improved to confirm these results. Furthermore, a double-blind design should reduce patient bias. Within future research lines, eyedrop manufacturer laboratories should be open to the option of producing personalized lubricants for each patient. Regarding dry eye disease pathophysiology, one or more tear layers will be affected, so a different excipient in eyedrop composition will be needed. In addition, a study with a population of fertile women with a higher age range would be interesting to correlate the effect of contraceptives and age. New variables could be included in future research analyses, such as osmolarity or matrix metalloproteinase (MMP-9). Therefore, the indications for dry eye disease treatment should include a thorough dry eye examination and evaluation of its causes.

## 5. Conclusions

Hyaluronic acid in combination with galacto-xyloglucan significantly decreased subjective dry eye disease symptoms and increased first and mean NIBUT compared to hyaluronic acid alone. Galacto-xyloglucan added efficacy in young-adult childbearing-age oral contraceptive users.

## Figures and Tables

**Table 1:** Demographics, invasive test, and subjective questionnaires before and after eyedrop treatment.

Variable	0.40% HA + 0.20% XG (*n* = 34)	0.40% HA (*n* = 34)	*p* Value
Demographics			
Age (years)	21.91 ± 0.98 (21.00 to 23.00)	21.59 ± 1.04 (20.00 to 23.00)	0.24
Sphere Refraction (D)	−1.17 ± 2.86 (-8.50 to +3.25)	−1.02 ± 1.48 (-3.50 to +1.00)	0.65
Cylinder Refraction (D)	−0.70 ± 0.91 (−3.75 to 0.00)	−0.48 ± 0.65 (−2.25 to 0.00)	0.15
Axis Refraction (Degrees)	95.86 ± 62.87 (2.00 to 170.00)	92.76 ± 65.00 (12.00 to 180.00)	0.55
CDVA (Decimal)	−1.07 ± 0.09 (1.00 to 1.20)	1.09 ± 0.10 (1.00 to 1.20)	0.53
Superior Eyelid MGD (Percentage)	26.40 ± 12.98 (5.40 to 49.40)	32.90 ± 5.42 (21.40 to 42.80)	0.05
Inferior Eyelid MGD (Percentage)	36.50 ± 15.96 (10.90 to 76.60)	31.61 ± 8.68 (19.30 to 45.40)	0.25
Invasive Test and Subjective Questionnaires Previous Treatment			
BUT (Seconds)	10.18 ± 3.12 (5.00 to 15.00)	7.94 ± 1.65 (5.00 to 11.00)	<0.01
OSDI (Score)	23.13 ± 13.15 (2.50 to 39.00)	27.26 ± 11.13 (12.00 to 52.00)	0.30
SPEED (Score)	8.97 ± 5.33 (0.00 to 14.00)	9.88 ± 2.84 (5.00 to 14.00)	0.81
Invasive Test and Subjective Questionnaires Posterior Treatment			
BUT (Seconds)	10.70 ± 2.22 (8.00 to 14.00)	8.71 ± 1.93 (6.00 to 12.00)	<0.01
OSDI (Score)	6.12 ± 3.64 (1.00 to 12.00)	15.65 ± 3.82 (10.00 to 22.00)	<0.01
SPEED (Score)	4.91 ± 1.75 (2.00 to 8.00)	9.18 ± 1.71 (6.00 to 12.00)	<0.01

HA: hyaluronic acid, GX: galacto-xyloglucan, D: diopter, CDVA: corrected distance visual acuity, MGD: meibomian gland dysfunction, BUT: break-up time, OSDI: Ocular Surface Disease Index, SPEED: Standard Patient Evaluation of Eye Dryness.

**Table 2:** Ocular surface analyzer comparison previous and after both six-week eyedrop treatments.

**Variable**		**0.40% HA + 0.20% GX (*n* = 34)**	**0.40% HA** **(*n* = 34)**	***p*** **Value**
Baseline	Conjunctival Redness (Efron Scale)	0.61 ± 0.49 (0.00 to 1.00)	0.68 ± 0.47 (0.00 to 1.00)	0.55
	Lipid Layer Thickness (Guillon Pattern)	1.64 ± 1.05 (0.00 to 4.00)	1.35 ± 0.81 (0.00 to 3.00)	0.39
	Tear Meniscus Height (Millimeters)	0.18 ± 0.04 (0.11 to 0.28)	0.19 ± 0.04 (0.13 to 0.25)	0.30
	First NIBUT (Seconds)	4.22 ± 1.12 (0.36 to 5.64)	4.48 ± 0.78 (3.07 to 5.93)	0.76
	Mean NIBUT (Seconds)	12.94 ± 5.22 (4.78 to 20.80)	11.36 ± 3.95 (5.21 to 18.04)	0.14
After six weeks	Conjunctival Redness (Efron Scale)	0.58 ± 0.50 (0.00 to 1.00)	0.47 ± 0.50 (0.00 to 1.00)	0.39
	Lipid Layer Thickness (Guillon Pattern)	1.52 ± 0.61 (0.00 to 2.00)	1.38 ± 0.65 (0.00 to 2.00)	0.38
	Tear Meniscus Height (Millimeters)	0.17 ± 0.03 (0.10 to 0.21)	0.18 ± 0.02 (0.12 to 0.22)	0.05
	First NIBUT (Seconds)	5.97 ± 0.57 (5.12 to 6.96)	5.03 ± 0.65 (4.02 to 6.23)	<0.01
	Mean NIBUT (Seconds)	16.66 ± 4.32 (7.34 to 21.70)	13.55 ± 2.59 (8.40 to 16.60)	<0.01

HA: crosslinked hyaluronic acid, GX: galacto-xyloglucan, NIBUT: non-invasive break-up time.

## Data Availability

The data presented in this study are available on request from the corresponding author. The data are not publicly available due to their containing information that could compromise the privacy of research participants.

## References

[1] Stapleton F., Alves M., Bunya V.Y., Jalbert I., Lekhanont K., Malet F., Na K.S., Schaumberg D., Uchino M., Vehof J. (2017). TFOS DEWS II Epidemiology Report. Ocul. Surf..

[2] Jones L., Downie L.E., Korb D., Benitez-del-Castillo J.M., Dana R., Deng S.X., Dong P.N., Geerling G., Hida R.Y., Liu Y. (2017). TFOS DEWS II Management and Therapy Report. Ocul. Surf..

[3] Rocha E.M., Mantelli F., Nominato L.F., Bonini S. (2013). Hormones and dry eye syndrome: An update on what we do and don’t know. Curr. Opin. Ophthalmol..

[4] Truong S., Cole N., Stapleton F., Golebiowski B. (2014). Sex hormones and the dry eye. Clin. Exp. Optom..

[5] Verjee M.A., Brissette A.R., Starr C.E. (2020). Dry Eye Disease: Early Recognition with Guidance on Management and Treatment for Primary Care Family Physicians. Ophthalmol. Ther..

[6] Sullivan D.A., Rocha E.M., Aragona P., Clayton J.A., Ding J., Golebiowski B., Hampel U., McDermott A.M., Schaumberg D.A., Srinivasan S. (2017). TFOS DEWS II Sex, Gender, and Hormones Report. Ocul. Surf..

[7] Auw-Haedrich C., Feltgen N. (2003). Estrogen receptor expression in meibomian glands and its correlation with age and dry-eye parameters. Graefe’s Arch. Clin. Exp. Ophthalmol..

[8] Suzuki T., Schirra F., Richards S.M., Jensen R.V., Sullivan D.A. (2008). Estrogen and progesterone control of gene expression in the mouse meibomian gland. Investig. Ophthalmol. Vis. Sci..

[9] Sullivan D.A., Jensen R.V., Suzuki T., Richards S.M. (2009). Do sex steroids exert sex-specific and/or opposite effects on gene expression in lacrimal and meibomian glands?. Mol. Vis..

[10] Suzuki T., Schirra F., Richards S.M., Treister N.S., Lombardi M.J., Rowley P., Jensen R.V., Sullivan D.A. (2006). Estrogen’s and progesterone’s impact on gene expression in the mouse lacrimal gland. Investig. Ophthalmol. Vis. Sci..

[11] Mostafa S., Seamon V., Azzarolo A.M. (2012). Influence of sex hormones and genetic predisposition in Sjögren’s syndrome: A new clue to the immunopathogenesis of dry eye disease. Exp. Eye Res..

[12] Pfeilschifter J., Köditz R., Pfohl M., Schatz H. (2002). Changes in proinflammatory cytokine activity after menopause. Endocr. Rev..

[13] Azzarolo A.M., Eihausen H., Schechter J. (2003). Estrogen prevention of lacrimal gland cell death and lymphocytic infiltration. Exp. Eye Res..

[14] Suzuki T., Sullivan D.A. (2005). Estrogen stimulation of proinflammatory cytokine and matrix metalloproteinase gene expression in human corneal epithelial cells. Cornea.

[15] He B., Iovieno A., Etminan M., Kezouh A., Yeung S.N. (2022). Effects of hormonal contraceptives on dry eye disease: A population-based study. Eye.

[16] Chen S.P., Massaro-Giordano G., Pistilli M., Schreiber C.A., Bunya V.Y. (2013). Tear osmolarity and dry eye symptoms in women using oral contraception and contact lenses. Cornea.

[17] Moschos M.M., Nitoda E. (2017). The impact of combined oral contraceptives on ocular tissues: A review of ocular effects. Int. J. Ophthalmol..

[18] Rivlin K., Isley M.M. (2018). Patient-centered Contraceptive Counseling and Prescribing. Clin. Obstet. Gynecol..

[19] Gomes J.A.P., Amankwah R., Powell-Richards A., Dua H.S. (2004). Sodium hyaluronate (hyaluronic acid) promotes migration of human corneal epithelial cells in vitro. Br. J. Ophthalmol..

[20] Fallacara A., Vertuani S., Panozzo G., Pecorelli A., Valacchi G., Manfredini S. (2017). Novel artificial tears containing cross-linked hyaluronic acid: An in vitro re-epithelialization study. Molecules.

[21] Aragona P., Di Stefano G., Ferreri F., Spinella R., Stilo A. (2002). Sodium hyaluronate eye drops of different osmolarity for the treatment of dry eye in Sjögren’s syndrome patients. Br. J. Ophthalmol..

[22] Serrano-Morales J.M., De-Hita-Cantalejo C., Sánchez-González M.C., Bautista-Llamas M.J., Sánchez-González J.M. (2022). Efficacy of 0.1% crosslinked hyaluronic acid, coenzyme Q10 and vitamin E in the management of dry eye disease in menopause patients receiving antidepressants. Eur. J. Ophthalmol..

[23] Uccello-Barretta G., Balzano F., Vanni L., Sansò M. (2013). Mucoadhesive properties of tamarind-seed polysaccharide/hyaluronic acid mixtures: A nuclear magnetic resonance spectroscopy investigation. Carbohydr. Polym..

[24] Kaur H., Yadav S., Ahuja M., Dilbaghi N. (2012). Synthesis, characterization and evaluation of thiolated tamarind seed polysaccharide as a mucoadhesive polymer. Carbohydr. Polym..

[25] Piqué N., Gómez-Guillén M.D.C., Montero M.P. (2018). Xyloglucan, a plant polymer with barrier protective properties over the mucous membranes: An overview. Int. J. Mol. Sci..

[26] Rolando M., Valente C. (2007). Establishing the tolerability and performance of tamarind seed polysaccharide (TSP) in treating dry eye syndrome: Results of a clinical study. BMC Ophthalmol..

[27] Molina-Solana P., de Borja Domínguez-Serrano F., Garrido-Hermosilla A.M., Montero-Iruzubieta J., Fernández-Palacín A., Rodríguez-De-la-rúa-franch E., Caro-Magdaleno M. (2020). Improved tear film stability in patients with dry eye after hyaluronic acid and galactoxyloglucan use. Clin. Ophthalmol..

[28] Tian L., Qu J.H., Zhang X.Y., Sun X.G. (2016). Repeatability and reproducibility of noninvasive keratograph 5m measurements in patients with dry eye disease. J. Ophthalmol..

[29] Nichols J.J., Nichols K.K., Puent B., Saracino M., Mitchell G.L. (2002). Evaluation of tear film interference patterns and measures of tear break-up time. Optom. Vis. Sci..

[30] Bandlitz S., Peter B., Pflugi T., Jaeger K., Anwar A., Bikhu P., Nosch D.S., Wolffsohn J.S. (2020). Agreement and repeatability of four different devices to measure non-invasive tear breakup time (NIBUT). Contact Lens Anterior Eye.

[31] Markoulli M., Duong T.B., Lin M., Papas E. (2018). Imaging the Tear Film: A Comparison Between the Subjective Keeler Tearscope-Plus^TM^ and the Objective Oculus^®^ Keratograph 5M and LipiView^®^ Interferometer. Curr. Eye Res..

[32] Fleischman D.S., Navarrete C.D., Fessler D.M.T. (2010). Oral contraceptives suppress ovarian hormone production. Psychol. Sci..

[33] Butovich I.A., Millar T.J., Ham B.M. (2008). Understanding and analyzing meibomian lipids—A review. Curr. Eye Res..

[34] Korb D.R., Scaffidi R.C., Greiner J.V., Kenyon K.R., Herman J.P., Blackie C.A., Glonek T., Case C.L., Finnemore V.M., Douglass T. (2005). The effect of two novel lubricant eye drops on tear film lipid layer thickness in subjects with dry eye symptoms. Optom. Vis. Sci..

[35] Fogt J.S., Kowalski M.J., King-Smith P.E., Epitropolous A.T., Hendershot A.J., Lembach C., Maszczak J.P., Jones-Jordan L.A., Barr J.T. (2016). Tear lipid layer thickness with eye drops in meibomian gland dysfunction. Clin. Ophthalmol..

[36] Scaffidi R.C., Korb D.R. (2007). Comparison of the efficacy of two lipid emulsion eyedrops in increasing tear film lipid layer thickness. Eye Contact Lens.

[37] Lim P., Han T.A., Tong L. (2020). Short-term changes in tear lipid layer thickness after instillation of lipid containing eye drops. Transl. Vis. Sci. Technol..

[38] Gipson I.K. (2004). Distribution of mucins at the ocular surface. Exp. Eye Res..

[39] Millar T.J., Tragoulias S.T., Anderton P.J., Ball M.S., Miano F., Dennis G.R., Mudgil P. (2006). The surface activity of purified ocular mucin at the air-liquid interface and interactions with meibomian lipids. Cornea.

[40] Pflugfelder S.C., Stern M.E. (2020). Biological functions of tear film. Exp. Eye Res..

[41] Barabino S., Rolando M., Nardi M., Bonini S., Aragona P., Traverso C.E. (2013). The effect of an artificial tear combining hyaluronic acid and tamarind seeds polysaccharide in patients with moderate dry eye syndrome: A new treatment for dry eye. Eur. J. Ophthalmol..

[42] Chun T., Maccalman T., Dinu V., Ottino S., Phillips-Jones M.K., Harding S.E. (2020). Hydrodynamic compatibility of hyaluronic acid and tamarind seed polysaccharide as ocular mucin supplements. Polymers.

[43] Cagini C., Di Lascio G., Torroni G., Mariniello M., Meschini G., Lupidi M., Messina M. (2021). Dry eye and inflammation of the ocular surface after cataract surgery: Effectiveness of a tear film substitute based on trehalose/hyaluronic acid vs hyaluronic acid to resolve signs and symptoms. J. Cataract Refract. Surg..

[44] Caretti L., Valerio A.L.G., Piermarocchi R., Badin G., Verzola G., Masarà F., Scalora T., Monterosso C. (2019). Efficacy of carbomer sodium hyaluronate trehalose vs hyaluronic acid to improve tear film instability and ocular surface discomfort after cataract surgery. Clin. Ophthalmol..

[45] Fogagnolo P., Romano D., De Ruvo V., Sabella P., Rossetti L. (2022). Clinical Efficacy of an Eyedrop Containing Hyaluronic Acid and Ginkgo Biloba in the Management of Dry Eye Disease Induced by Cataract Surgery. J. Ocul. Pharmacol. Ther..

[46] Mencucci R., Favuzza E., Decandia G., Cennamo M., Giansanti F. (2021). Hyaluronic acid/trehalose ophthalmic solution in reducing post-cataract surgery dry eye signs and symptoms: A prospective, interventional, randomized, open-label study. J. Clin. Med..

[47] De-Hita-Cantalejo C., Sánchez-González M.C., Silva-Viguera C., García-Romera M.C., Feria-Mantero R., Sánchez-González J.M. (2022). Efficacy of hyaluronic acid 0.3%, cyanocobalamin, electrolytes, and P-Plus in menopause patients with moderate dry eye disease. Graefe’s Arch. Clin. Exp. Ophthalmol..

[48] Kiss H.J., Németh J., Jhanji V. (2015). Isotonic glycerol and sodium hyaluronate containing artificial tear decreases conjunctivochalasis after one and three months: A self-controlled, Unmasked study. PLoS ONE.

